# Temporal transcriptomic response during arsenic stress in *Herminiimonas arsenicoxydans*

**DOI:** 10.1186/1471-2164-11-709

**Published:** 2010-12-17

**Authors:** Jessica Cleiss-Arnold, Sandrine Koechler, Caroline Proux, Marie-Laure Fardeau, Marie-Agnès Dillies, Jean-Yves Coppee, Florence Arsène-Ploetze, Philippe N Bertin

**Affiliations:** 1 UMR7156 Université de Strasbourg/CNRS, Génétique Moléculaire, Génomique et Microbiologie, Département Microorganismes, Génomes, Environnement, 28 rue Goethe, 67083 Strasbourg cedex, France; 2 Plateforme technologique Puces à ADN, Institut Pasteur, 28 rue du Dr. Roux, 75724 Paris cedex 15, France; 3 IRD Laboratoire de Microbiologie, ESIL, 163 Avenue de Luminy 13288 Marseille cedex 9, France

## Abstract

**Background:**

Arsenic is present in numerous ecosystems and microorganisms have developed various mechanisms to live in such hostile environments. *Herminiimonas arsenicoxydans*, a bacterium isolated from arsenic contaminated sludge, has acquired remarkable capabilities to cope with arsenic. In particular our previous studies have suggested the existence of a temporal induction of arsenite oxidase, a key enzyme in arsenic metabolism, in the presence of As(III).

**Results:**

Microarrays were designed to compare gene transcription profiles under a temporal As(III) exposure. Transcriptome kinetic analysis demonstrated the existence of two phases in arsenic response. The expression of approximatively 14% of the whole genome was significantly affected by an As(III) early stress and 4% by an As(III) late exposure. The early response was characterized by arsenic resistance, oxidative stress, chaperone synthesis and sulfur metabolism. The late response was characterized by arsenic metabolism and associated mechanisms such as phosphate transport and motility. The major metabolic changes were confirmed by chemical, transcriptional, physiological and biochemical experiments. These early and late responses were defined as general stress response and specific response to As(III), respectively.

**Conclusion:**

Gene expression patterns suggest that the exposure to As(III) induces an acute response to rapidly minimize the immediate effects of As(III). Upon a longer arsenic exposure, a broad metabolic response was induced. These data allowed to propose for the first time a kinetic model of the As(III) response in bacteria.

## Background

Bacteria live in changing environments and are subjected to a variety of environnmental stresses such as pH, temperature, osmolarity or heavy metals. Arsenic is found in numerous disturbed or natural ecosystems where it can exist in mutiple oxidation states, the most common being arsenite As(III) and arsenate As(V) [1]. This metalloid is known to generate oxidative stress in cells by its capability to induce the formation of reactive oxygen species (ROS). The damages caused by ROS to lipids, proteins and DNA are likely to contribute to As toxicity [2,3]. In addition, one property of arsenite, which indicates that it behaves like a soft metal, consists in a high reactivity with sulphydryls groups and that affects the activity of many proteins. Microorganisms have developed remarkable capabilities to cope with arsenic. The most common arsenic resistance mechanism depends on the presence on plasmid or chromosome of *ars *genes encoding proteins involved in the reduction and/or the efflux of the toxic element [4]. Nevertheless, other arsenic resistance mechanisms have been described, i.e. arsenite oxidation and arsenic methylation [5,6]. In addition, in various microbial species, arsenic stress is associated with arsenite oxidase activity, biofilm formation, motility, oxidative stress or sulfur assimilation [7,8]. For example, the biofilm development by *Thiomonas arsenivorans *has been described as a physical barrier decreasing As(III) access to sessile cells [9]. Remarkably, some organisms such as *Rhizobium sp*. NT-26, have evolved specific metabolic pathways allowing them to oxidize As(III) as an energy source [10,11] or others are known to use As(V) in anaerobic respiration [12]. In the heterotrophic prokaryote *Herminiimonas arsenicoxydans*, genome sequencing revealed the presence of four *ars *operons involved in the reduction of As(V) to As(III) and of one *aoxAB *operon involved in the oxidation of the most toxic form, As(III) to the less toxic form As(V) [13]. In addition to this detoxification process, this bacterium exhibits positive chemotaxis and motility towards arsenic, and metalloid scavenging by exopolyssacharides.

The availability of the *H. arsenicoxydans *complete genome sequence offers an opportunity to study its physiology by functional genomic approaches [13]. Our previous transcriptomic studies have demonstrated that a large number of genes encoding proteins involved in oxidative stress, low affinity import of phosphate or DNA repair are induced after 15 min As(III) exposure. However, no variation was found in the genes coding for arsenite oxidase, a key enzyme in arsenic response [14] recently shown to be subjected to a complex regulation [15]. In addition, little is known regarding the kinetics of arsenic stress response in microorganisms. To address these processes, physiological analyses coupled with Western immunoblotting experiments and DNA microarrays were used to examine the temporal changes in transcriptome profiles during the transition from As(III) to As(V) species due to As(III) oxidation. Our work represents, to our knowledge, the first kinetic analysis of transcription pattern in bacteria exposed to arsenic, leading to propose a global model of arsenic response in *H. arsenicoxydans*.

## Results and Discussion

### Characterization of arsenic oxidoreduction kinetics

**Figure 1:**
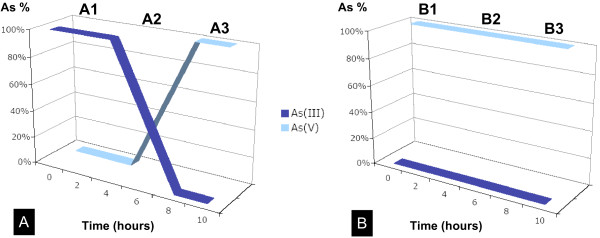
**Temporal dynamics of arsenic oxidoreduction in *H. arsenicoxydans***. (A) Time course measurement of As(III) and As(V) species after As(III) exposure.

To study the oxidoreduction kinetic in *H. arsenicoxydans*, the two arsenic species (As(III) and As(V)) were quantified by HPLC-ICP-AES from filtered culture supernatants at several times after As(III) or As(V) induction (0, 15 min, 1, 2, 4, 6 and 8 hours) (Figure 1). In the presence of As(III) (Figure 1A), three distinct phases were observed: in phase A1 (early exposure: 0 to 4 hours), no oxidation was observed since arsenic was present as 100% As(III). In phase A2, which corresponds to a partial oxidation of As(III) into As(V), both species were present, a proportion of 50% As(III) and 50% As(V) being observed after 6 hours. The transformation of As(III) into As(V) was complete after 8 hours (phase A3).

### Overview of transcriptome profiles

To get insight into the mechanisms involved in arsenic response, including oxidoreduction processes, transcriptomic analyses were performed at different steps of the experimental time course. Cells were collected from cultures of *H. arsenicoxydans *induced 15 min (early), 6 hours (intermediary), 8 hours (late) or not with 0,66 mM As(III) (Figure 1A) or 13,3 mM As(V) (Figure 1B). These concentrations were choosen as they are far from *H. arsenicoxydans *MIC for As(III) or As(V), which is able to grow up to 5 mM and 100 mM of As(III) and As(V), respectively (Muller, 2007). Moreover, this concentration of As(III) had only a minor impact on growth rate and cells continued to divide immediately after its addition. Under As(V) stress, 128 genes were up-regulated and 107 genes were down-regulated in the early phase (B1) (7% of the genome). In the late phase (B2) the number of genes up-regulated (239 genes) and down-regulated (161 genes) increased (12% of the genome). Under As(III) stress, the proportion was inverted. The major shift in gene expression occured during the onset of the early phase (A1) with 214 genes up-regulated and 245 genes down-regulated under As(III) stress. This represents approximatly 14% of the genes whose expression was significantly affected by As(III). As cell growth progressed further into the late phase (A3), the number of genes up-regulated (60 genes) and down-regulated (73 genes) decreased, representing 4% of the genome. The large number of genes responding to As(III), especially in the early phase, suggests a major remodelling of cellular physiology. In addition, oxidoreduction kinetic was only observed under As(III) stress (Figure 1A and 1B), suggesting that the Aox-dependent oxidation largely predominate the Ars-dependent reduction process. This study therefore focused on the As(III) response in *H. arsenicoxydans*.

### Hierachical clustering kinetics

**Figure 2:**
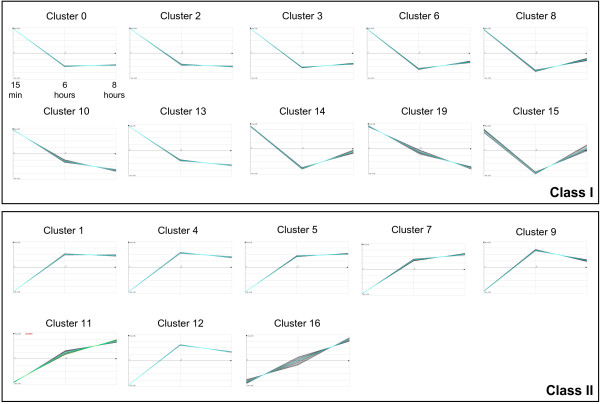
**Patterns of gene expression changes during As(III) exposure**. Expression graphs of the 20 clusters obtained by plotting the log_2 _fold-change values (y-axis) at each of the three time points (x-axis). The average for all genes in each cluster is highligthed by a blue line. Class I and Class II contained genes whose expression levels gradually decreased or increased after the induction, respectively.

**Figure 3:**
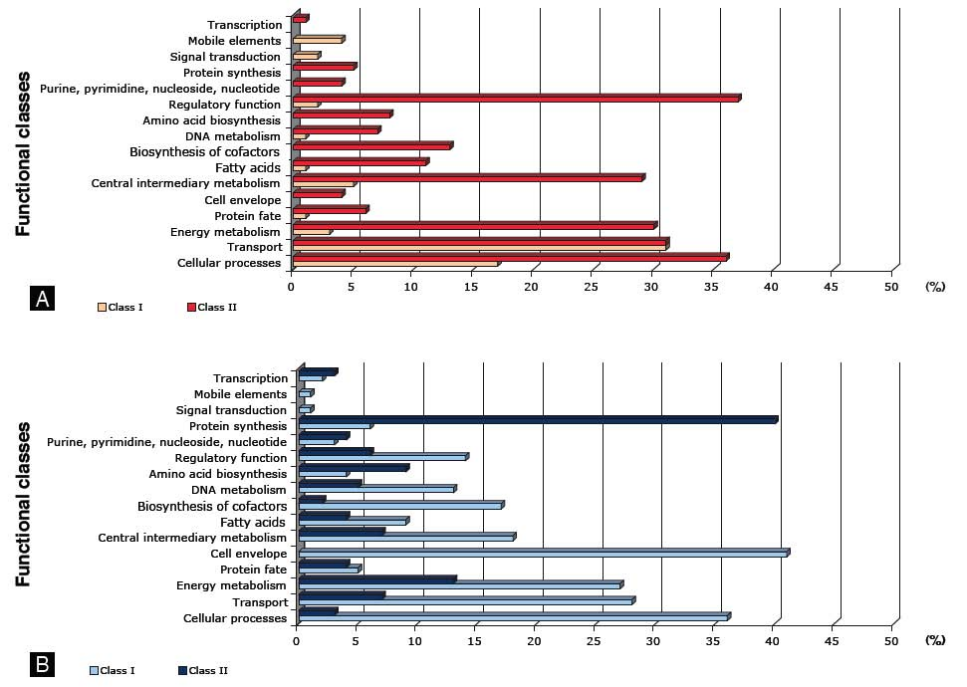
**Distribution of genes differentially expressed under arsenic stress and specific to Class I or II associated in clusters of functional categories**. (A) induced and (B) repressed.

To classify the transcripts on the basis of their temporal dynamics after exposure to arsenite, we performed a hierarchical clustering with the ArrayMiner^® ^software http://www.optimaldesign.com/ArrayMiner/ArrayMiner.htm. Genes were sorted into groups that exhibited similar transcription kinetics as cells transitioned from A1 to A3 phases. The set of genes regulated by As(III) shown in Figure 2 was classified into 20 clusters. The profiles similarity of those clusters enabled us to identify two major classes: 1) class I: (clusters 0, 2, 3, 6, 8, 10, 13, 14, 15 and 19) containing genes whose expression increased immediately after 15 min induction and decreased after 6 and 8 hours and class II: (clusters 1, 4, 5, 7, 9, 11, 12 and 16) containing genes whose expression levels increased only after 6 and 8 hours induction. The product of each upregulated or downregulated gene by a fold change of two or more was identified to gain insight into the changes in physiology [Additional file 1: Supplemental Table S1]. Next, to examine how the two classes are distributed with regard to their functions, we further classified these genes according to the Gene Classification based on COG functional categories (Figure 3). The overall gene expression pattern suggests the existence of two major physiologic phases (early and late) during the adaptation to As(III) stress.

#### i) Early phase

Our microarray analysis highlighted a set of stress responsive genes whose transcription was early affected by As(III). This phase contained the early up-regulated genes whose expression levels gradually decreased after the induction (Class I) or down-regulated genes whose expression levels gradually increased after the induction (Class II).

First, σ^E ^and several genes encoding proteins involved in the synthesis of cell envelope components were down-regulated (Class II, Figure 3B (regulatory function, protein synthesis and fatty acid)) whereas genes encoding proteins involved in transport were up-regulated (Class I, Figure 3A (transport)). The bacterial envelope is a major defense against threats from the environment. Due to its crucial importance, the integrity of the cell envelope, including the presence of outer membrane proteins (OMP), is closely monitored to ensure its functionality. The σ^E ^system is the major pathway [16] required for proper folding of OMPs and their turnover [17], phospholipids and lipopolysaccharide (LPS) biosynthesis, signal transduction and the expression of inner and outer membrane proteins [18]. In *Escherichia coli*, the *σ*^E ^regulon is induced specifically in response to the unbalanced synthesis of outer membrane proteins [17] and to the misfolding of polypeptides that have been translocated across the cytoplasmic membrane [19]. Our results suggest that both the organization (lipoproteins, peptidoglycan and LPS) and the functioning (transport, signal transduction) of the bacterial envelope may be changed in the early phase of the response to As(III).

Second, early As(III) stress strongly stimulated expression of genes encoding components of sulfur assimilation, sulfur oxidation and glutathione (GSH) biosynthesis pathways. In fact, mRNA levels of genes encoding all the enzymatic steps required for sulfate uptake, its conversion into cysteine and GSH were increased. The expression of these genes was induced about 3 to 22 fold after 15 min induction with As(III). The expression of some of these genes was down-regulated at 6 and 8 hours [Additional file 1: Supplemental Table S1, Class II]. In eukaryotic cells, As(III) exposure results in increased levels of reduced glutathione [20] and an As(III)-triglutathione complex has been found in human liver excreta [21], leading to the conclusion that GSH plays an important role in reducing As(III) toxicity. Most effects of arsenite result from its interaction with thiol groups of proteins, its iniophoretic properties and its ability to directly or indirectly generate free radicals and hence induce oxidative stress [22]. Microorganisms have therefore developed defense mechanisms that either keep the concentration of the O_2_-derived radicals at acceptable levels or repair oxidative damages. In our previous proteomic experiments, oxidative stress proteins, such as KatA and SodB, were shown to be induced in response to As(III) stress in *H. arsenicoxydans *[14]. Similarly, we observed in the present study a positive regulation of three genes (*sodC, katA, ahpC*) involved in the protection against oxidative stress. The *sodC *gene encodes the superoxide dismutase catalyzing the dismutation of the superoxide radical to H_2_O_2_, which is less toxic, and O_2_. In addition, *katA *and *ahpC *genes encoding a catalase and a peroxidase, respectively, are responsible for scarvenging endogenously produced H_2_O_2_. Finally, As(III) early exposure induced a glutaredoxin and various thioredoxin encoding transcripts [Additional file 1: Supplemental Table S1]. In *H. arsenicoxydans *the induction of genes encoding proteins involved in sulfate and GSH pathways suggests that GSH may play a key role in the cell defense against oxidative stress and metalloid toxicity by maintaining an intracellular reducing environment. GSH may detoxify arsenite by i) chelation and then removing of arsenite from the cell, ii) protection against oxidation caused by metals since GSH serves as the main redox buffer in the cell, and iii) binding to reactive sulfhydryl groups of proteins thereby protecting them from irreversible metalloid binding and/or oxidative damages [23]. Thioredoxin is believed to serve as a cellular antioxidant by maintaining the intracellular redox state, which is greatly influenced by the presence or absence of intracellular ROS, by reducing disulfide bonds produced by various oxidants and by interacting with proteins by direct association or thiol reactivity. Glutaredoxin is also known as a thioltransferase and like thioredoxin possesses an active center disulphide bond. Finally, arsenic could indirectly produce ROS by inactivating catalytic iron-sulfur clusters of enzymes as it has been shown for enzymes of the deshydratase family in *E. coli *in the presence of copper. In this organism, iron-sulfur clusters are the primary targets of copper and the resulting damages lead to the release of iron atoms in the cell and the generation of hydroxyl radicals [24]. All these cellular activities (GSH, catalase, peroxidase and thioredoxin-related) are thought to be part of a general set of reactions involved in the direct or indirect response to As(III)-mediated production of reactive oxygen species in *H. arsenicoxydans*.

Third, the adaptive responses of bacteria to sudden environmental changes, usually involve the induction of many major heat shock proteins MHSPs, including chaperones, proteases, transcriptional regulators and other structural proteins. As(III) induced the expression of several genes encoding MHSPs [Additional file 1: Supplemental Table S1]. The MHSPs play a role in repairing and preventing damages caused by an accumulation of unfolded proteins resulting from diverse environmental stresses. In agreement with this, our microarray data showed that As(III) early exposure induced the transcription of two *uspA *genes that might be involved in protecting bacterial cells against As(III) stress. The protein UspA has previously shown to be induced by As(III) [14]. The expression of the universal stress protein A (UspA) is known to be induced by a large variety of stress conditions [25], our results show that these conditions include As(III).

Finally, arsenic resistance in *H. arsenicoxydans *is partially mediated by proteins encoded by three different *ars *operons and one *aox *operon [13]. The clusters of *ars *genes encode an ArsR regulator, an As(III) extrusion pump ArsB/Acr3, an ArsH putative flavoprotein and one or two arsenate reductases ArsC. One of the loci contains an Acr3-type transporter, the others associate an ArsB-type transporter with ArsC reductases. ArsC is an enzyme that reduces As(V) to As(III) which can be pumped out by the ArsB or Acr3 membrane proteins. Both *acr3 *and *arsB *genes were induced in the presence of As(III). However, Acr3 mRNAs were only detected in 15 min As(III) exposed cells, which demonstrates that Acr3 is specific to the early response. Remarkably, the arsenite oxidase genes were not induced in early phase. The lack of induction of the *aox *genes is in agreement with the detection by HPLC-IC-AES of 100% As(III) (Figure 1).

#### ii) Late phase

This phase contains genes whose expression levels gradually increased (class II) or decreased (class I), after arsenic exposure. Multiple genes of cellular processes, cell envelope and metabolism (energy, intermediary, DNA...) were highly downregulated in class I (Figure 3B). Class II was characterized by the induction of several functional categories, i.e. genes involved in metabolism, regulatory functions and cellular processes (Figure 3A). These genes are suggested to be specific for the late response to As(III).

First, *H. arsenicoxydans *reacted to a late As(III) exposure by activating genes involved in the transport and assimilation of phosphate as well as other phosphorous compounds [Additional file 1: Supplemental Table S1]. Indeed, after late exposure, phosphate specific transporter (Pst) and phosphate inorganic transporter (Pit) were induced. Although we cannot exclude that the various genes involved in phosphate uptake were strongly induced because of a partial depletion of phophate in the medium, this induction could also result from the accumulation of As(V). Indeed, As(V) produced by the oxidation of As(III), which only occurred in late phase (Figure 1), may compete with phosphate because of their structural similarity. So, *H. arsenicoxydans *may preferentially transport phosphate *via *the specific Pst phosphate transport system rather than the Pit general transport mechanism, in order to reduce the entry of As(V). These observations suggest that a complex regulation of the *pst *and *pit *operons allows *H. arsenicoxydans *to maintain its intracellular phosphate level despite the accumulation of As(V). This further supports our previous hypothesis on the physiological link between arsenic and phosphate metabolism [13].

Second, genes involved in chemotactic machinery and flagellum assembly were up-regulated after late exposure to As(III). Our results from the microarray experiments during exposure to As(III) combined to previous observations [13] clearly demonstrate that the chemotactic response is induced by As(III). In *H. arsenicoxydans *the flagellar machinery is organized in a mixed peritrichous/polar cascade which is most probably synthesized through sequential hierarchy of gene activation events initiated by the expression of the master transcriptional regulator FlhDC [13]. The microarray results strongly suggest that motility is gradually induced in response to As(III) in *H. arsenicoxydans*.

Finally, the *aox *operon was induced after late exposure. The *aox *operon contains *aoxA *encoding the small subunit of arsenite oxidase, *aoxB *encoding the large subunit, *aoxC *encoding a nitroreductase and *aoxD *encoding a cytochrome c552. The *aoxRS *operon is involved in the regulation of *aoxABCD *operon [15]. All of them were induced by late As(III) stress [Additional file 1: Supplemental Table S1, Class II], which strongly suggests that arsenite oxidase activity is specific to late exposure.

### Transcriptional and physiological analysis of the major metabolic changes

Our microarray data suggest that several genes play a major role in the transition from the absence of As(III) to early or late arsenic exposure. To confirm this hypothesis, chemical, transcriptional, physiological and biochemical experiments were performed.

First, to further support the involvement of glutathione synthesis and sulfate metabolism in early As(III) response (early phase) in *H. arsenicoxydans*, we performed quantitative RT-PCR experiments. Transcripts abundance of *gloA *and *soxC *were compared to two internal controls, i.e the putative RNA methyltransferase gene and the peptide deformylase gene, in cultures challenged 15 min, 8 hours or not by As(III). The expression of *soxC *and *gloA *mRNA was increased by a 312 and 7 fold factor, respectively, after 15 min As(III) exposure. No such induction was observed in late exposure.

To address the possible role of As(III) in the GSH pathway, the total content of GSH was measured. The pool of GSH increased after 15 min (~3 fold) and further rose over time (5 fold after 6 hours). After 8 hours the level remained unchanged, as compared with 6 hours. These observations suggest that the accumulation of GSH in response to As(III) was specific to the early phase. In addition, although we did not observed any change in the extracellular concentration of sulfate, genes encoding sulfate uptake were up-regulated after 15 min and down regulated after 6 and 8 hours [Additional file 1: Supplemental Table S1]. Moreover, genes involved in the sulfur oxidation pathway, i.e. *soxC, soxD*, were also induced [Additional file 1: Supplemental Table S1]. A similar induction of enzymes involved in sulfur metabolism has already been shown under oxidative stress in *Pseudomonas aeruginosa *[26]. Oxidative stress enzymes, i.e. thioredoxin and superoxide dismutase, are known to be implicated in sulfur assimilation and metabolism [27,28]. Our results suggest that the intracellular concentration of different species of sulfur compounds is altered in *H. arsenicoxydans *under arsenic stress, demonstrating for the first time that sulfur metabolism is also impaired in response to arsenic stress.

On the other hand, to confirm the oxidative stress response resulting from arsenic exposure, peroxidase activity was measured from cultures induced or not, 15 min, 6, 8 hours with As(III). The activity was induced 5 fold after 15 min and remained unchanged after late exposure. This observation demonstrates that peroxidase activity is also specific to the early exposure to As(III).

**Figure 4:**
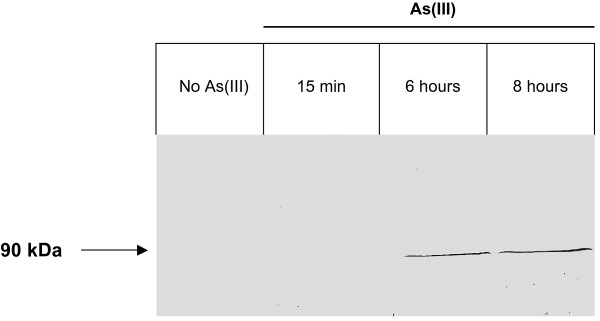
**Immunodetection of AoxB protein in total protein extracts of *H. arsenicoxydans *induced or not for 15 min, 6 or 8 hours with As(III)**. AoxB was detected as a single band at 90 kDa.

Second, the induction of arsenite oxidase activity in the late phase was demonstrated in this study (Figure 1). The oxidation was complete after 8 hours exposed cells. On agar plate, the activity was detectable after 48 hours growth in presence of As(III), by a silver nitrate staining test (data not shown). The accumulation of arsenite oxidase was tested by Western immunoblotting experiments with anti-AoxB antibodies. AoxB was detected in protein extract from cells induced for 6 and 8 hours. In contrast, no protein was detected after 15 min induction (Figure 4) which explains the lack of *aox *gene expression after 15 min in the presence of As(III) observed in a previous study [14]. Arsenite oxidase activity is therefore specific to late exposure to As(III) in *H. arsenicoxydans*. In addition, in As(III) non exposed cultures of some bacteria, As(III) oxidation did not occur until cultures reached stationary phase, as suggested in *Agrobacterium tumefaciens *[29], indicating that quorum sensing is involved in the regulation of As(III) oxidation. However, in *H. arsenicoxydans*, no significant induction of genes involved in quorum sensing was observed in the time course experiment.

Similarly, to support the effect of arsenic on flagellar motility and phosphate transport in the late phase, the transcript abundance of *fliC *and *pstSb *was measured. *pstSb *mRNAs increased by a 455 fold factor after 8 hours As(III) exposure but no such induction was observed in early exposure. Remarkably, *fliC *was induced by a 14 and 11 fold change after 15 min and 8 hours, respectively.

**Figure 5:**
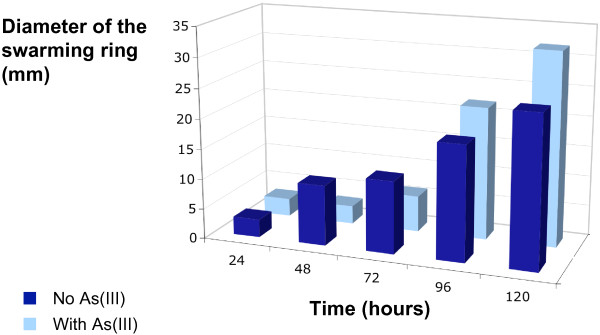
**Effect of temporal exposure to arsenite on *H. arsenicoxydans *motility**. The level of motility at 24, 48, 72, 96 and 120 hours was evaluated as the diameter of the swarming ring in mm. The results presented are the mean value of five independent experiments.

The motility of *H. arsenicoxydans *was analyzed in the presence and the absence of As(III). As shown in Figure 5 a swarming diameter of 10 mm was measured for cells incubated 48 hours whithout arsenic on the semi-solid medium. In the presence of As(III), cells were mobile on semi-solid medium only after 72 hours incubation (12 mm), which corresponds to the late phase in liquid medium. These results suggest that the presence of As(III) delays cell motility. However, no significant difference in the expression of flagellar genes was observed between early and late exposure [Additional file 1: Supplemental Table S1]. Moreover, the expression of *motA *and *motB *genes, encoding proteins responsible for the rotary activity of flagellum motor [30], was not affected by early or late exposure to As(III). These observations clearly demonstrate that the flagellum biosynthesis was not altered in response to As(III), unlike its functioning. In *H. arsenicoxydans*, the rotation of this flagellar appendange is driven by a sodium motive force [13]. Because the arsenite oxidase is known to be a periplamic enzyme, in agreement with Western immunoblotting experiments on cell fractions (data not shown), and the oxidation of arsenite releases four protons [31], we proposed that arsenite oxidase activity may indirectly increase motility. Indeed, a Na^+^/H^+ ^antiporter has been shown to be involved in *A. tumefaciens *As(III) oxidation [32]. In *H. arsenicoxydans*, the genome exploration revealed the possible synthesis of such a Na^+^/H^+ ^transporter. The protons released from arsenite oxidation may thus extrude Na^+ ^through this antiporter and generate the electron potential for the Na^+^-driven flagellar motor, as described in the differentiation of flagellar system of *Vibrio alginolyticus *[33]. This hypothesis could explain how arsenite oxidase contributes to the motility of *H. arsenicoxydans *after As(III) late exposure.

## Conclusion

**Figure 6:**
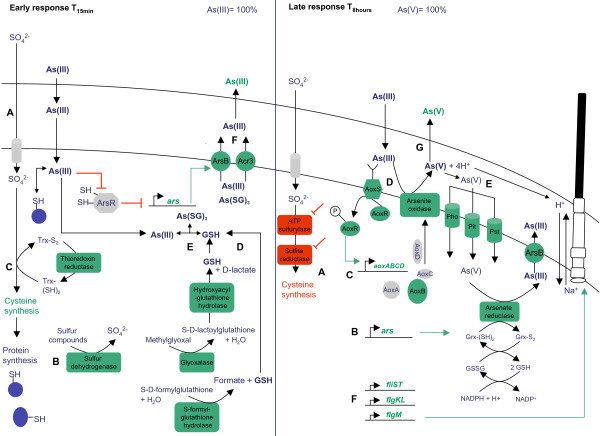
**Conceptual representation of the metabolic response after temporal exposure to As(III) in *H. arsenicoxydans***. (A) after 15 min induction (early phase) and (B) after 8 hours induction (late phase). Genes repressed or induced after As(III) exposure are shown with a red or green background, respectively.

Many microorganisms have acquired the capacity to survive in the presence of toxic metals or metalloids by inducing the expression of resistance genes. The specific nature of these resistance mechanisms is the result of a cleverly designed genetic circuit that is tightly controlled by regulatory proteins. However, the multiple physiological responses induced by arsenite stress in *H. arsenicoxydans *are not exclusively associated with the expression of classical arsenic resistance operons. Indeed, in this study we demonstrated that the adaptation to As(III) is characterized by an early and a late response. A highly sophisticated regulatory network may thus modulate the expression of genes in response to arsenic. These effects are mediated in part through the activation or repression of mRNA transcripts by DNA-binding proteins and signal transduction systems, such as ArsR, AoxRS and others. All these transcriptomic observations are summarized in a kinetic and metabolic model (Figure 6).

The early response is characterized by the induction of genes involved in general stress. First, GSH is a protagonist of the primary cellular responses to As(III). The genes encoding enzymes involved in sulfate uptake (A), sulfur oxidation (B), cysteine synthesis (C) leading to cysteine containing proteins and their conversion in GSH (D) were induced. As(III)-exposed cells may thus channel a large part of the assimilated sulfur into GSH for metal chelation, cellular redox buffering and possibly also protein glutathionylation. As(III) may react with GSH to form an As(SG)_3 _complex (E). Interactions of these As-thiol group non specific complexes with molecular oxygen lead to the formation of reactive oxygen species such as H_2_O_2_, resulting in oxidative stress within the cell. The As(III) is extruded by ArsB or Acr3 (F) and Acr3 induction was demonstrated to be specific to early exposure. The mechanism proposed here is in agreement with the observations made with HPLC-ICP-AES, showing that no oxidation occurred in early As(III) stress and only As(III) species was present.

The late response was characterized by the induction of genes specific for arsenic. Indeed, the GSH response is downregulated (A) and the transcripts encoding arsenic resistance (B), especially arsenite oxidase and its regulation (*aoxRS*) (C, D), and associated mechanisms such as phosphate uptake (E) and flagellum biosynthesis (F) were upregulated. The arsenite oxidase catalyses the oxidation of As(III) in As(V) (D). As(V) may directly enter the cell via the Pst or Pit transporter (E), where it is reduced to As(III) by the arsenate reductase and extruded into the periplasm *via *ArsB (F). Then As(V) produced by the oxidation of As(III) is extruded out of the cell (G). The mechanism proposed here for the late response to As(III) is in agreement with the oxidoreduction kinetics and underlies the links between arsenic, phosphate and motility.

In this study, we demonstrated that the detoxification and metabolism processes are gradually expressed in *H. arsenicoxydans *to adapt to arsenic-rich environments. In bacteria, the response to As(III) seems therefore to be characterized by a stereotypic just-in-time transcription of specific metabolic pathways, resulting in a two-step response.

## Methods

### Bacterial strains and growth media

*H. arsenicoxydans *ULPAs1 was grown in a chemically defined medium (CDM), supplemented by 2% agar when required [34]. Tryptone swarm plates containing CDM supplemented with 1% Bacto-Tryptone and 0.25% agar were used to assess bacterial motility.

### Arsenic speciation and sulfate determination

*H. arsenicoxydans *was grown in CDM medium and cultures were induced when required by addition of 0.66 mM As(III) for 15 min, 1 hour, 2, 4, 6 or 8 hours. Similarly, cultures were induced or not for 15 min, 6 hours, 8 hours with 13.3 mM As(V). Culture supernatants were filtered through sterile 0,22 μm pore size filters (VWR). Arsenic species were separated by high-performance liquid chromatography (HPLC) and quantified by inductively coupled plasma-atomic emission spectrometry (ICP-AES), as previously described [35]. Sulfate extracellular concentration was determined by ion chromatography (IC) using a Metrohm Compact IC 761 system. The column used was a MetrosepA SUPP 1-250 (4.6+250 mm) with an eluent concentration of 3 mM sodium carbonate.

### Peroxidase and total glutathione assay

Peroxidase activity and total glutathione were measured from serially diluted (10^0^-10^-3^) 48 hours-old cultures of *H. arsenicoxydans *exposed or not to As(III). The peroxidase activity was determined using the Amplex Red Hydrogen Peroxide Assay kit (Invitrogen) and the total glutathione concentration was measured using the colorimetric glutahione detection kit (PromoKine). This assay was performed according to the protocol provided by the manufacturer.

### Preparation of protein extracts, SDS-PAGE separation

Protein extracts and Western immunoblotting experiments were performed as described previously in Koechler *et al. *[15]

### RNA extraction

Strains were grown at 25°C for 24 hours (OD = 0,15 at 600 nm, corresponding to log-phase cells) and cultures were induced or not by addition of 0,66 mM As(III) for 15 min, 6 hours or 8 hours before extraction. After 8 hours induction the OD was checked and was still corresponding to log-phase cells (OD = 0,56). Similarly, 13,3 mM As(V) were added or not to a 24 hours cultures during 15 min, 6 hours or 8 hours. Samples were harvested and stored at - 80°C. RNA were extracted as previously described [14]. After extraction procedure, RNA integrity was checked by electrophoregram analysis on a BioAnalyser (Agilent) and total RNA concentration was determined spectrophotometrically with a Nanodrop.

### Microarrays and data analysis

Microarrays containing 60-mer oligonucleotides for all predicted *H. arsenicoxydans *genes http://www.genoscope.cns.fr/agc/mage/arsenoscope were used, as previously described [14]. Briefly, total RNA (5 μg) was reverse transcribed and indirectly labelled and next hybridized to the arrays. Three distinct biological RNA samples were prepared from each growth condition at different time after induction with As(III) or As(V) (0 min and after 15 min, 6 hours or 8 hours) and labelled either by Cy3 or Cy5 in a dye-swap design. Microarray data were deposited in ArrayExpress (accession E-MEXP-2809). Scanning, data normalization and statistical analysis was performed as described [14]. Genes showing a valid *p*-value and a more than two-fold decreased or increased expression were considered as differentially expressed between the conditions tested and were retained for further study.

Clustering analysis was performed using ArrayMiner http://www.optimaldesign.com/ V. 5.3.4 with the Gaussian clustering and Pearson correlation options selected. The data table included log2 ratios at three different time points (15 min, 6 and 8 hours) with t = 0 being a reference. 2349 genes were significantly up- or down-regulated in at least one of the three time points. The number of classes has to be tuned prior to the run. It was chosen to 20, as it appeared to provide homogeneous profiles within classes with a reasonable number of unclassified genes.

### Quantitative real time PCR

Quantitative PCR experiments were performed as described by Koechler *et al. *[15], with the following modification of thermocycling conditions: 5 min at 95°c and 40 cycles of 15 s at 95°C, 15 s at 60°c and 1 min at 72°C. Two biological replicates (independent cultures) and two quantitative PCR replicates were performed in each experience. Amplification products were designed in order to obtain less than 175 bp fragments. The pairs of primers used are listed in Additional file 1: Supplemental Table S1.

## Authors' contributions

JCA and SK wrote the manuscript. JCA performed the microarray data and clustering analysis, Western immunoblotting and motility experiments, arsenic, peroxidase and total glutathione determination. SK carried out the quantitative PCR experiments. CP, MAD and JYC conceived and performed the transcriptomic and clustering experiments. MLF determined the sulfate concentration. FP and PB helped to analyse the data and critically revised the manuscript. PB coordinated and conceived the study. All authors read and approved the final manuscript.

## Supplementary Material

Additional file 1 **Supplemental table S1**. Selected genes differentially expressed after 15 min or 8 hours arsenite stress. Genes are classified according to their class and cluster. Class I contains genes whose expression increased after 15 min induction and decreased after 8 hours. Class II contains genes whoses expression increased only after 8 hours induction. Click here for file
