# Supplementary material for: Temporal transcriptomic response during arsenic stress in Herminiimonas arsenicoxydans
Source: BMC Genomics. 2010 Dec 17;11:709. doi: 10.1186/1471-2164-11-709 (PMC3022917; doi:10.1186/1471-2164-11-709)
Supplement: Additional file 1 — Supplemental table S1. Selected genes differentially expressed after 15 min or 8 hours arsenite stress. Genes are classified according to their class and cluster. Class I contains genes whose expression increased after 15 min induction and decreased after 8 hours. Class II contains genes whoses expression increased only after 8 hours induction. [file 1471-2164-11-709-S1.PDF]

CLASS I

CLUSTER 0

			As(III) 15 min	As(III) 8 hours	
HEAR0028	*ahpF*	Alkyl hydroperoxide reductase subunit F (Alkyl hydroperoxide reductase F52A protein)	23,9		Cell. proc
HEAR0040		putative outer membrane efflux protein	2,1		Transport
HEAR0041		putative secretion proteinHlyD	2,7		Transport
HEAR0182		Putative thioredoxin	5,1		Energy met
HEAR0196		putative metallopeptidase M61 family	4,0		Protein fate
HEAR0213		Putative Integral membrane protein TerC family	3,7		Cell env
HEAR0370	*gloA*	S-D-lactoylglutathione methylglyoxal lyase (glyoxalase I)	3,7		Cent. Int. met
HEAR0642	*thiL*	thiamin-monophosphate kinase	2,1		Biosynthesis cof.
HEAR0649		putative 5'-nucleotidase	2,2		DNA met
HEAR0754	*argD*	acetylornitine transaminase	3,4		Aa biosynthesis
HEAR0764	*eco*	ecotin precursor	2,1		Protein fate
HEAR0862		Protein TldD homolog	2,4		Cell. proc
HEAR0863	*aro*	Phospho-2-dehydro-3-deoxyheptonate aldolase	2,2		Aa biosynthesis
HEAR1041	*ubiD*	3-octaprenyl-4-hydroxybenzoate decarboxylase	3,0		Biosynthesis cof.
HEAR1190	*bcp*	Pseudoazurin precursor (Blue copper protein)	12,0		
HEAR1232		conserved hypothetical protein ; putative polyhydroxyalkanoate granule associated protein	6,3		Reg. function
HEAR1363	*norA*	regulator of cell morphogenesisand NO signaling	3,0		Reg. function
HEAR1394		Putative glycine cleavage T protein (aminomethyltransferase)	4,4		Protein fate
HEAR1429		putative transport protein (MFS family)	37,5		Cell env
HEAR1430	*nemA*	N-ethylmaleimide reductase, FMN-linked	38,3		Cell. proc
HEAR1457		Putative transcription elobgation factor GreA	8,8		Reg. function
HEAR1467		Putative tripartite ATP-independent periplasmic transporter DctQ	4,1		Transport
HEAR1481	*folE*	GTP cyclohydrolase I (GTP-CH-I)	4,9		Biosynthesis cof.
HEAR1617		transcriptional regulator (CadR)	12,9		Reg. function
HEAR1747	*tesA*	acyl-CoA thioesterase I precursor (Protease I)	2,5		Fatty acid
HEAR1962	*acr3*	Arsenite efflux pump ACR3	25,3		Cell. proc
HEAR1997		conserved hypothetical protein, putative competence protein	2,6		Cell. proc
HEAR2093		putative ABC type transporter system, permease component	2,6		Transport
HEAR2125		HAM1 protein homolog	2,8		Pur. Pyr. Ns. Nts
HEAR2239	*iscS*	Cysteine desulfurase	5,8		Cent. Int. met
HEAR2240	*iscR*	Iron-sulfur cluster assembly transcription factor IscR	7,6		Reg. function
HEAR2390	*cysB*	HTH-type transcriptional CysB (Cys regulon transcriptional activator)	2,5		Aa biosynthesis
HEAR2429	*aptA*	Omega-amino acid--pyruvate aminotransferase	3,6		Cent. Int. met
HEAR2466	*ispH*	4-hydroxy-2-methylbut-2-enyl-diphosphate reductase	2,5		Biosynthesis cof.
HEAR2668		Putative RNA methylase	3,8		DNA met
HEAR2670	*gabD*	succinate-semialdehyde dehydrogenase	4,4		Cent. Int. met
HEAR2696		putative Two-component response regulator	2,3		Reg. function
HEAR2713	*acrA2*	component of acridine efflux pump, AcrA-like	11,5		Cell. proc
HEAR2714	*acrB2*	Acriflavine resistance protein B	3,0		Cell. proc
HEAR2722		Putative formyl CoA transferase Frc	5,1		Cent. Int. met
HEAR2723	*croA*	linoyl-CoA hydratase	10,5		Fatty acid
HEAR2724		Putative amino acid aldolase/racemase	10,9		Cent. Int. met
HEAR2727		conserved hypothetical membrane protein	6,2		Transport
HEAR2731	*sgaA*	Serine--glyoxylate aminotransferase	31,8		Cent. Int. met
HEAR2733		putative D-lactate dehydrogenase	3,6		Energy met
HEAR2839	*hslR*	heat shock protein homolog	3,5		Cell. proc
HEAR2927		putative 3-methyladenine DNA glycosylase	2,6		DNA met
HEAR2933		Putative permease	2,5		Transport
HEAR2990		Putative Type III pantothenate kinase (CoaX-like)	2,0		Reg. function
HEAR3132		putative CYTOCHROME C assembly protein	2,6		Biosynthesis cof.
HEAR3315		putative deoxygenase	2,2		Cent. Int. met
HEAR3324		putative regulator protein	11,3		Reg. function
HEAR3325		Putative transporter component domain	10,5		Transport
HEAR3327	*frdB*	Succinate dehydrogenase/fumarate reducatse Fe-S subunit	5,4		Energy met
HEAR3328		putative fumarate reductase respiratory complex transmembrane subunit	4,5		Energy met
HEAR3332		putative tartrate dehydratase	3,1		Energy met
HEAR3336		ABC transporter, ATP binding protein transport	35,6		Transport
HEAR3337	*uxaA*	Altronate dehydratase	17,8		Cent. Int. met
HEAR3339		ABC Transporter , protein component	3,3		Transport
HEAR3450		PutativeMFS permease-like protein (C part)	2,0		Transport
HEAR3454		Luciferase-like monooxygenase	2,9		Cell. proc
HEAR3458	*tkrA*	Putative phosphoglycerate dehydrogenase	5,7		Cent. Int. met
HEAR2499	*cysP*	Thiosulfate-binding protein precursor		-2,6	Transport
HEAR1811		Putative ADP-ribose pyrophosphate		-2,2	Pur. Pyr. Ns. Nts
HEAR1457		Putative transcription elongation factor GreA		-3,2	Reg. function
HEAR0850	*gap*	glyceraldehyde 3-phosphate dehydrogenase		-2,2	Cent. Int. met

HEAR3172	*rpoC*	DNA-directed RNA polymerase subunit beta' (RNAP subunit beta') (Transcriptase subunit beta') (RNA polymerase subunit beta')		-3,3	Transcription

CLUSTER 2

HEAR0013		putative NADPH dehydrogenase	4,3		Energy met
HEAR0034		putative transcription elongation factor GreB	6,4		Reg. function
HEAR0048	*norZ*	Nitric oxide reductase	3,5		Energy met
HEAR0121	*lrp*	Leucine-responsive regulatory protein	3,0		Reg. function
HEAR0122	*rocD*	Ornithine aminotransferase (Ornithine--oxo-acid aminotransferase)	4,3		Aa biosynthesis
HEAR0123		Conserved hypothetical protein ; putative maidinotransferase	3,1		Aa biosynthesis
HEAR0204	*apaG*	Protein ApaG	2,0		Transport
HEAR0244		conserved hypothetical protein ; putative protein CcmA	2,5		Cell env.
HEAR0245	*orpA*	Iron binding protein ErpA (Iron-sulfur cluster insertion protein)	3,1		Cent. Int. met
HEAR0298		Conserved hypothetical protein ; putative nucleoside-diphosphate sugar epimerase	2,5		Cent. Int. met
HEAR0675		putative transcriptional regulator aminotransferase GntR	2,1		Biosynthesis cof.
HEAR0818		putative transcriptional regulator (IclR family)	2,5		Reg. function
HEAR0853		Conserved hypothetical protein ; putative alkylphosphonate uptake protein PhnA	2,1		Transport
HEAR0917		putative Hydroxyacylglutathione hydrolase	7,3		Cent. Int. met
HEAR0919		Putative fumarate hydratase class I	2,2		Energy met
HEAR0978		putative sulfate permease (SulP family)	2,0		Cell env
HEAR1044	*aidB*	Isovalenyl coA dehydrogenase	2,9		Cell. proc
HEAR1185		putative Beta lactamase,soxH homolog	10,8		Cell. proc
HEAR1188	*soxC*	SoxC protein	21,9		Energy met
HEAR1189		Cytochrome c precursor (putative SoxD)	19,8		Energy met
HEAR1193	*soxA*	Putative cytochrome c SoxA	7,4		Energy met
HEAR1374	*cydA*	cytochrome d ubiquinol oxidase subunit I	4,6		Energy met
HEAR1405	*moaA*	molybdopterin biosynthesis, protein A	2,5		Biosynthesis cof.
HEAR1455		two-component response regulator protein, LuxR-family	2,0		Reg. function
HEAR1460	*smpD2*	SsrA-binding protein	2,4		Protein synthesis
HEAR1466		Putative TRAP dicarboxylate transporter-DctP subunit	5,6		Transport
HEAR1482		Cytochrome c	4,2		Energy met
HEAR1484		putative RNA polymerase sigma factor	8,1		Reg. function
HEAR1489		Putative 3 demethyl ubiquinne -9 3-methyl-transferase	2,6		Biosynthesis cof.
HEAR1519		conserved hypothetical protein, putative lambda repressor-like, DNA binding	4,2		Reg. function
HEAR1539		Putative cointegrate resolution protein S (TnpS protein)	2,8		DNA met
HEAR1705		ABC type transport system involved in resistance to inorganic solvents, permease component	2,6		Transport
HEAR1725		putative transcriptional regulator, AraC family	2,4		Reg. function
HEAR1788		putative ABC type molybdate transport system, periplasmic component ModA	4,1		Transport
HEAR1805	*bdhA*	D-beta-hydroxybutyrate dehydrogenase	2,2		Fatty acid
HEAR1808		TRANSCRIPTION REGULATOR LuxR family	2,7		Reg. function
HEAR2039		Alcohol dehydrogenase class III (Glutathione-dependent formaldehyde dehydrogenase) (FDH) (FALDH)	2,8		Cent. Int. met
HEAR2044		putative transcriptional regulator LysR family	2,2		Reg. function
HEAR2047		putative transcription regulator, LysR family	2,4		Reg. function
HEAR2146	*moaE*	molybdopterin converting factor, subunit 2	2,2		Cell. proc
HEAR2147	*moaD*	Molybdopterin converting factor subunit	2,4		Biosynthesis cof.
HEAR2148	*moeA2*	molybdopterin biosynthesis protein MoeA	2,6		Biosynthesis. cof
HEAR2471		Putative transcriptional regulator	2,7		Reg. function
HEAR2523		Putative ribonuclease BN	2,3		Transcription
HEAR2526	*fumC*	fumarate hydratase Class II fumarase C)	2,1		Cent. Int. met
HEAR2584	*ubiG*	3-demethylubiquinone-9 3-methyltransferase	3,2		Biosynthesis cof.
HEAR2601	*guaC*	GMP reductase	3,8		Cent. Int. met
HEAR2683		putative regulatory protein IclR family	2,5		Reg. fucntion
HEAR2837	*yusL*	Putative bifunctional protein 3-hydroxyacyl-CoA dehydrogenase and dodecenoyl-CoA isomerase	2,4		Fatty acid
HEAR2908	*rpoH*	RNA polymerase sigma 32 factor	4,9		Biosynthesis cofactors
HEAR3266		Putative methylisocitrate lyase (PrpB)	3,9		Cent. Int. met
HEAR3267	*prpC*	2 methylcitrate synthase (citrate synthase 2)	3,6		Cent. Int. met
HEAR3271		Putative OsmC like protein	3,8		Cell. proc
HEAR3288		putative RIBONUCLEASE BN	2,6		Transcription
HEAR3318		putative 2-nitropropane dioxygenase	2,1		Energy met
HEAR3330		putative fumarate dehydratase	4,4		Energy met
HEAR3338		ABC transporter ATP binding domain	4,6		Transport

HEAR3359		putative MS CS Mechanosensitive ion channel	2,4		Cell. proc
HEAR3433	*fdsB*	NAD dependent formate dehydrogenase beta subunit	2,0		Energy met
HEAR1035	*leuA1*	2-isopropylmalate synthase		-2,7	Aa biosynthesis
HEAR2136	*metE*	5-methyltetrhydropteroyl triglutamate-homocystéine methyltransferase		-2,6	Aa biosynthesis
HEAR2950	*sahH*	Adenosylhomocysteinase (S-adenosyl-L-homocysteine hydrolase) (AdoHcyase)		-4	Aa biosynthesis
HEAR0442		Putative RNA polymerase sigma factor, sigma70 factor *rpoD*		-2,2	Reg. function
HEAR1086	*edd*	6-phosphogluconate dehydratase		-2,1	Energy met

CLUSTER 3

HEAR0026		putative transcriptional regulatory protein with PAS domain	2,7		Reg. function
HEAR0072	*sugE*	Quaternary ammonium compound-resistance protein sugE	3,2		Cell. proc
HEAR0239		Putative OsmC-like protein	2,1		Cell. proc
HEAR0282		putative transcription regulator protein, LysR family	2,4		Reg. function
HEAR0308		putative transcriptional regulator, MarR family	2,4		Reg. function
HEAR0311		putative cytochrome b561	5,4		Energy met
HEAR0614		gamma-glutamyltranspeptidase	3,5		Cent. Int. met
HEAR0756		putative nucleoside-diphosphate-sugar epimerases	4,2		Pur. Pyr. Ns. Nts
HEAR0798		putative NADH dehydrogenase/NAD(P)H nitroreductase RutE	2,1		Pur. Pyr. Ns. Nts
HEAR0916		putative transcriptional regulator (ArsR family)	11,3		Reg. function
HEAR0983		putative transcription regulators (MarR family)	5,4		Reg. function
HEAR0984		putative RND efflux system outer membrane lipoprotein NodT	6,5		Transport
HEAR0985	*emrA*	multidrug resistance protein A	3,5		Cell. proc
HEAR1089	*nadA*	quinolinate synthetase A	2,1		Biosynthesis cof.
HEAR1090	*nadC*	nicotinate-nucleotide pyrophosphorylase (quinolinate phosphoribosyltransferase)	2,1		Biosynthesis cof.
HEAR1205		Flavodoxin/nitricoxide synthase	2,5		Reg. function
HEAR1448		conserved hypothetical protein ; putative membrane protein	4,8		Cell. proc
HEAR1449		putative multiple antibiotic resistance protein MarC	2,2		Cell. proc
HEAR1465		transcriptional regulatory protein ; GntR family	2,3		Reg. function
HEAR1764		putative peptidase M22, glycoprotease	2,1		Protein fate
HEAR2036	*frmB*	S-formylglutathione hydrolase	2,2		Cent. Int. met
HEAR2040	*frnR*	Repressor FrmR	2,6		Reg. function
HEAR2237	*iscA*	Iron-binding protein IscA (iron-sulfur assembly protein)	3,1		Protein synthesis
HEAR2238	*iscU*	Fe-S cluster assembly scaffold IscU	4,1		Protein synthesis
HEAR2401	*pepA*	Cytosol aminopeptidase	2,6		Protein fate
HEAR2432	*rbfA*	ribosome-binding factor A	2,3		Protein synthesis
HEAR2524		conserved hypothetical protein ; putative stress response protein	2,8		Cell. proc
HEAR2585	*gph*	phosphoglycolate phosphatase PGP 2	2,1		Cent . Int. met
HEAR2664	*proA*	gamma-glutamylphosphate reductase	2,6		Aa biosynthesis
HEAR2667		putative DSBA oxidoreductase	2,3		Protein fate
HEAR2711	*acrR*	Putative transcriptional repressor (acrR) for multidrug efflux pump	13,0		Cell. proc
HEAR2729		putative GntR-family transcriptional regulator	8,2		Reg. function
HEAR2899	*fdx2*	Ferredoxin	2,1		Energy met
HEAR2952	*metK*	methionine adenosyltransferase adenosylmethionine synthetase MAT	5,4		Cent. Int. met
HEAR2973	*grxC*	glutaredoxin 3 Grx3	3,0		Energy met
HEAR3236		putative permease	2,3		Transport
HEAR3242		putative permease of the major facilitator superfamily	2,9		Transport
HEAR3329		putative fumarate reductase	3,1		Energy met
HEAR3340		putative Phosphoglycerate dehydrogenase	2,1		Aa biosynthesis
HEAR3378	*ectC*	L-ectoine synthase (N-acetyldiaminobutyrate dehydratase)	2,4		Cell. proc
HEAR3379	*ectB*	Diaminobutyrate--2-oxoglutarate aminotransferase (L- diaminobutyric acid transaminase) (Diaminobutyrate transaminase)	2,7		Cent. Int. met
HEAR3380	*ectA*	L-2,4-diaminobutyric acid acetyltransferase (DABA acetyltransferase)	3,4		Cent. Int. met
HEAR3467	*icd2*	isocitrate dehydrogenase	2,2		Energy met
HEAR3143	*rpsK*	30S ribosomal subunit protein S11		-7,7	Protein synthesis
HEAR2485		Putative lactate permease		-2,1	Transport
HEAR2948	*metF*	5,10-methylenetetrahydrofolate reductase		-2,3	Aa biosynthesis
HEAR1166	*greA*	Transcription elongation factor		-2,3	Reg. function
HEAR2392	*cysI*	Sulfite reductase		-2,9	Cent. Int. Met
HEAR2394	*cysH*	Phosphoadenosine phosphosulfate reductase (PAPS reductase, thioredoxin dependent) (PAdoPS reductase) (3'- phosphoadenylylsulfate reductase) (PAPS sulfotransferase)		-2,6	Cent. Int. met
HEAR2395	*cysD*	Sulfate adenylyltransferase subunit 2 (Sulfate adenylate transferase) (SAT) (ATP-sulfurylase small		-3,1	Cent. Int. Met

		subunit)			
HEAR2396	*cysN*	Sulfate adenylyltransferase subunit 1 (Sulfate adenylate transferase) (SAT) (ATP-sulfurylase large subunit)		-3,4	Cent. Int. met
HEAR2952	*metK*	S-adenosylmethionine synthetase (Methionine adenosyltransferase) (AdoMet synthetase) (MAT)		-2,4	Cent. Int. met
HEAR1813	*nuoN*	NADH-ubiquinone oxidoreductase, chain N		-3,4	Energy met
HEAR1814	*nuoM*	NADH-quinone oxidoreductase subunit M		-4	Energy met
HEAR1815	*nuoL*	NADH-quinone oxidoreductase subunit L (NADH dehydrogenase I subunit L) (NDH-1 subunit L)		-4,3	Energy met
HEAR1816	*nuoK*	NADH-quinone oxidoreductase subunit K (NADH dehydrogenase I subunit K) (NDH-1 subunit K)		-4,3	Energy met
HEAR1817	*nuoJ*	NADH-quinone oxidoreductase subunit J (NADH dehydrogenase I subunit J) (NDH-1 subunit J)		-2,3	Energy met
HEAR1818	*nuoI*	NADH-quinone oxidoreductase subunit I (NADH dehydrogenase I subunit I) (NDH-1 subunit I)		-3,7	Energy met
HEAR1819	*nuoH*	NADH-quinone oxidoreductase subunit H (NADH dehydrogenase I subunit H) (NDH-1 subunit H)		-2,6	Energy met
HEAR3173	*rpoB*	DNA-directed RNA polymerase subunit beta (RNAP subunit beta) (Transcriptase subunit beta) (RNA polymerase subunit beta)		-3,7	Transcription

CLUSTER 6

HEAR0029	*ahpC*	Alkyl hydroperoxide reductase subunit C (Peroxiredoxin) (Thioredoxin peroxidase) (Alkyl hydroperoxide reductase protein C22) (SCRP-23) (Sulfate starvation- induced protein 8) (SSI8)	10,9		Cell. proc
HEAR1214		Putative permease M48	2,5		Protein fate
HEAR1538		Putative cointegrate resolution protein T	2,3		DNA met
HEAR2236	*hscB*	co-chaperone protein HscB (Hsc20)	2,5		Protein synthesis
HEAR2715	*oprM3*	outer membrane protein oprM	2,6		Cell. proc
HEAR2775		Putative cytochrome c assembly protein	2,0		Transport
HEAR2928		Putative two-component system regulatory protein	2,1		Reg. function
HEAR2574	*rpsA*	30S ribosomal subunit protein S1		-6,7	Protein synthesis
HEAR2894	*rplY*	50S ribosomal protein L25 (General stress protein CTC)		-6,7	Protein synthesis
HEAR3140		50S ribosomal protein L17		-5,3	Protein synthesis
HEAR3142	*rpsD*	30S ribosomal subunit protein S4		-5,3	Protein synthesis
HEAR3144	*rpsM*	30S ribosomal subunit protein S13		-5,6	Protein synthesis
HEAR3147	*rplO*	50S ribosomal subunit protein L15		-8,3	Protein syntheis
HEAR3148	*rpmD*	50S ribosomal subunit protein L30		-10	Protein synthesis
HEAR3149	*rpsE*	30S ribosomal subunit protein S5		-11,1	Protein synthesis
HEAR3157	*rpsQ*	30S ribosomal subunit protein S17		-9,1	Protzin synthesis
HEAR3158		50S ribosomal protein L29		-11,1	Protein synthesis
HEAR3159	*rplP*	50S ribosomal subunit protein L16		-10	Protein synthesis
HEAR3162	*rpsS*	30S ribosomal subunit protein S19		-11,1	Protein synthesis
HEAR3167	*rpsJ*	30S ribosomal subunit protein S10		-6,7	Protein synthesis
HEAR3176	*rplA*	50S ribosomal subunit protein L1		-6,7	Protein synthesis
HEAR3177	*rplK*	50S ribosomal subunit protein L11		-7,1	Protein synthesis
HEAR0871	*argG*	argininosuccinate synthetase (Citrulline—aspartate ligase)		-2,5	Aa biosynthesis
HEAR2150	*hom*	Homoserine dehydrogenase		-2,3	Aa biosynthesis
HEAR2577	*tyrA*	Prephenate dehydrogenase		-2	Aa biosynthesis
HEAR2578	*hisC2*	Histidinol phosphateaminotransferase		-3,1	Aa biosynthesis
HEAR0343	*sucC*	succinyl-CoA synthetase, beta subunit		-2,4	Cent. Int. met
HEAR1820	*nuoG*	NADH-quinone oxidoreductase subunit G (NADH dehydrogenase I subunit G) (NDH-1 subunit G)		-3,8	Energy met
HEAR3404	*atpC*	ATP synthase epsilon chain (ATP synthase F1 sector epsilon subunit)		-3,7	Energy met
HEAR3405	*atpD*	ATP synthase subunit beta (ATPase subunit beta) (ATP synthase F1 sector subunit beta)		-3,2	Energy met
HEAR2573	*ihfB*	Integration host factor subunit beta (IHF-beta)		-7,7	DNA met
HEAR3141	*rpoA*	RNA polymerase, alpha subunit		-5,2	Transcription

CLUSTER 8

HEAR1961		putative permease of facilitator superfamily	6,4		Transport
HEAR2391		putative permease	2,7		Transport
HEAR0703	*rpmE2*	50S ribosomal protein L31 type B		-4,8	Protein synthesis
HEAR0845	*ileS*	isoleucine tRNA synthetase (Isoleucine—tRNA ligase) (IleRS)		-2,4	Protein synthesis
HEAR1336	*tsf*	Elongation factor Ts (EF-Ts)		-3,7	Protein synthesis
HEAR2159	*rplI*	50S ribosomal subunit protein L9		-4,8	Protein synthesis
HEAR2160		30S ribosomal protein S18		-7,7	Protein synthesis
HEAR2433	*infB*	Translation initiation factor IF-2		-2,3	Protein synthesis
HEAR3145	*infA*	Translation initiation factor IF-1		-5,6	Protein synthesis
HEAR3146	*secY*	Preprotein translocase SecY subunit		-4,5	Transport
HEAR3406	*atpG*	ATP synthase gamma chain (ATP synthase F1 sector gamma subunit)		-3,1	Energy met
HEAR0844	*lspAB*	Lipoprotein signal peptidase (Prolipoprotein signal peptidase) (Signal peptidase II) (SPase II)		-2,7	Protein fate

Cluster 10

HEAR0143	*glnK*	Nitrogen regulatory protein P-II 2	2,6		Reg. function
HEAR0214		Putative Universal stress protein UspA	6,3		Cell. proc
HEAR0225	*gltI*	Glutamate/aspartate periplasmic binding protein precursor	2,1		Transport
HEAR0578	*phbB*	acetoacetylCoA reductase	2,2		Energy met
HEAR0684	*alpA*	prophage CP4-57regulatory protein AlpA	2,0		Reg. function
HEAR0883		putative Guanine deaminase (Guanase) (Guanine aminase) (Guanine aminohydrolase) (GAH) (GDEase)	2,9		Pur. Pyr. Ns. Nts
HEAR1187		putative Bacterial regulatory protein, arsR family	4,4		Reg. function
HEAR1200	*katA*	catalase (hydroperoxidase II)	4,3		Cell. proc
HEAR1371		putative cytochrome c precursor	2,9		Energy met
HEAR1907	*ompR1*	Transcriptional regulatory protein OmpR	2,2		Reg. function
HEAR1923	*phbC*	Poly-beta-hydroxybutyrate polymerase (Poly(3-hydroxybutyrate) polymerase) (PHB polymerase) (PHB synthase) (Poly(3- hydroxyalkanoate) polymerase) (PHA polymerase) (PHA synthase) (Polyhydroxyalkanoic acid synthase)	2,5		Energy met
HEAR2142	*putA*	Bifunctional protein putA proline dehydrogenase delta-1 pyrroline-5-carboxylate dehydrogenase (proline oxidase) (P5C dehydrogenase)	2,5		Aa biosynthesis
HEAR2682		putative leucine-, isoleucine-, valine-, threonine-, and alanine binding protein precursor BraC	2,5		Transport
HEAR2703		putative membrane transport protein, MFS family	3,4		Transport
HEAR3183		Putative thioredoxin	2,2		Cell. proc
HEAR3241		putative 4-oxalocrotonate tautomerase	4,6		Cent. Int. met
HEAR3297	*copA3*	copper transporting P-type ATPase CopA	5,8		Cell. proc
HEAR0316		Bacterioferritin bfr (cytochrome b 557.5)		-2,2	Cell. proc

Cluster 13

HEAR0186	*hemA*	Glutamyl-tRNA reductase (GluTR)	2,1		Biosynthesis cof.
HEAR0518		Putative transcription regulatory protein (Cad Rand PbrR)	2,9		Reg. function
HEAR0615	*upgQ*	Glycerophosphodiester phosphodiesterase	2,0		Cent. Int. Met
HEAR0840	*sodC*	Superoxide dismutase [Cu-Zn]	4,0		Cell. proc
HEAR0904		Putative universal stress protein family UspA	3,0		Cell. proc
HEAR1191	*soxY*	Putative Sulfur oxidation protein SoxY	6,8		Energy met
HEAR1192		Putative sulfur oxidation protein SoxZ	6,7		Energy met
HEAR1194		Putative cytochrome c (SoxX)	5,0		Energy met
HEAR1589		Putative cointegrate resolution protein (TnpS protein)	2,3		DNA met
HEAR1590		putative transcription regulator protein (CadR and PbrR family)	3,0		Reg. function
HEAR1610		Zinc containing alcool dehydrogenase superfamily	8,0		Cent. Int. met
HEAR1727		ATP-dependent DNA ligase precursor	2,1		DNA met
HEAR1806	*scoB*	succinyl-CoA:acetoacetyl-CoA transferase, subunit B	3,7		Fatty acid
HEAR1807	*scoA*	Succinyl-CoA:3-ketoacid-coenzyme A transferase subunit A	3,8		Fatty acid
HEAR1809		putative 3-oxoacyl-(acyl-carrier-protein) reductase	3,4		Fatty acid
HEAR2332		Conserved hypothetical protein, putative catalytic LigB subunit of armating ring-opening dioxygenase	2,3		Energy met
HEAR2415		putative osmotically inducible protein Y precursor OsmY-like	3,2		Cell. proc
HEAR2553	*opuD*	Glycine betaine transporter	2,8		Transport
HEAR2770	*nrdA*	ribonucleoside diphosphate reductase, alpha subunit	2,2		Cent. Int. met
HEAR2782	*nudH*	Putative (di)nucleotide polyphosphate hydrolase NudH-like	2,0		Cell. proc
HEAR2836	*fadA*	3-keto acyl CoA	2,4		Fatty acid
HEAR2838	*acdB*	Putative butyrylCoA dehydrogenase	3,4		Fatty acid
HEAR3432	*fdsA*	NAD dependent formate dehydrogenase, alpha subunit	2,3		Energy met

Cluster 14

HEAR3392	*ibp*	small heat-shock protein Hsp20	4,9		Cell. proc
HEAR2162	*rpsF*	30S ribosomal subunit protein S6		-3,3	Protein synthesis
HEAR2397	*cobA2*	Uroporphyrinogen-III C-methyltransferase		-2,3	Biosynthesis cof.
HEAR2078	*plsX*	Fatty acid/phospholipid synthesis protein PlsX		-2,1	Fatty acid
HEAR2161	*priB*	Primosomal replication protein N		-5	Cell. proc
HEAR2785		Conserved hypothetical protein, utative GTPase		-2,1	Cell. proc

Cluster 15

HEAR1501		Putative Fe2+ transport system protein A FeoA	2,6		Transport
HEAR2230	*lysS*	Lysyl-tRNA synthetase (Lysine—tRNA ligase) (LysRS)		-2,1	Protein synthesis
HEAR0305		putative Preprotein translocase subunit YajC		-2,2	Transport

HEAR2893	*prs*	Ribose-phosphate pyrophosphokinase (RPPK) (Phosphoribosyl pyrophosphate synthetase) (P-Rib-PP synthetase) (PRPP synthetase)		-2,3	Pur. Pyr. Ns. Nts
HEAR3072		Putative stress induced morphogen BolA protein		-2,1	Reg. function

CLUSTER 19

HEAR0039		putative Transcriptional regulator, TetR family	2,0		Reg. function
HEAR0105		small permease DctQ component	2,4		Transport
HEAR0106		putative TRAP-type C4-dicarboxylate transport system, periplasmic component Dct subunit	3,6		Transport
HEAR0221	*fadD*	Long-chain-fatty-acid--CoA ligase (Long-chain acyl-CoA synthetase)	2,1		Fatty acid
HEAR1583		Putative copper binding protein precursor	2,7		Cell. proc
HEAR1584		conserved hypothetical protein, Thioredoxin domain	2,7		Energy met
HEAR1959	*arsH*	NADPH-dependent FMN reductase	6,4		Cell. proc
HEAR2259		putative Cyclopropane-fatty-acyl-phospholipid synthase	4,1		Fatty acid
HEAR0387		Putative ABC type branched chain amino acid transport systems		-2,1	Transport

CLASS II

CLUSTER 1

HEAR0477	*aoxC*	putative nitroreductase		11,3	Cell. proc
HEAR0478	*aoxB*	Arsenite oxidase large subunit (AOI)		32,0	Cell. proc
HEAR0479	*aoxA*	Arsenite oxidase small subunit precursor , Rieske type subunit, twin arginine translocation peptide		54,9	Cell. proc
HEAR0481		putative phosphite transport system-binding protein PtxB precursor		3,7	Transport
HEAR0489		putative ABC-type phosphate transport system, auxiliary and permease component		48,8	Transport
HEAR0490	*pstB2*	Phosphate import ATP-binding protein		60,6	Transport
HEAR0491		putative Phosphate uptake regulator PhoU		72,5	Transport
HEAR0496		putative serine phosphatase		25,9	Reg. function
HEAR0581		Putative methyl-accepting chemotaxis protein		22,0	Cell. proc
HEAR1098		putative ABC-type branched-chain amino acid transport systems, periplasmic component		14,6	Transport
HEAR1102	*gtrA*	Glycosyltransferase		41,4	Cent. Int. met
HEAR1105		putative phosphohistidine phosphatase *sixA*		15,5	Signal transduction
HEAR1106	*ppk*	polyphosphate kinase (Polyphosphoric acid kinase) (ATP- polyphosphate phosphotransferase)		11,3	Cent. Int. met
HEAR1107	*pstS2*	Phosphate-binding periplasmic protein precursor (PBP)		16,6	Transport
HEAR1110	*pstB2*	Phosphate import ATP-binding protein PstB (Phosphate- transporting ATPase) (ABC phosphate transporter)		11,8	Transport
HEAR1111	*phoU2*	Phosphate transport system protein PhoU		12,6	Transport
HEAR1112	*phoB2*	Phosphate regulon transcriptional regulatory protein PhoB		9,7	Transport
HEAR1113	*phoR2*	Phosphate regulon sensor protein PhoR		4,3	Transport
HEAR1389		putative shikimate transporter		3,5	Transport
HEAR1866	fliC	flagellin		5,0	Cell. proc
HEAR1867		Putative flagellar protein FlaG		4,0	Cell. proc
HEAR3212	*ptxB*	phosphonate transport system substrate-binding protein precursor		19,8	Transport
HEAR3214	*ptxD*	Phosphonate dehydrogenase (NAD-dependent phosphite dehydrogenase)		14,9	Transport
HEAR3215	*ptxE*	Putative HTH-type transcriptional regulator protein PtxE-like		7,0	Transport
HEAR1504		Putative peptidase S11 D-analyl-D-alanine carboxypeptidase 1	-2,6		Cell env
HEAR2788	*ispB*	octaprenyl diphosphate synthase	-2,5		Biosynthesis cof.
HEAR2534		Putative permease of the MFS	-2		Transport
HEAR0378	*corC*	Magnesium and Cobalt efflux protein CorC	-2		Transport
HEAR1349	*rnhB*	Ribonuclease HII (RNAse HII)	-5,6		Transcription
HEAR1347	*lpxA*	Acyl-(acyl-carrier-protein) UDP-N-acetylglucosamine acetyltransferase	-2,9		Cell env
HEAR1437		Putative metallo-beta-lactamase	-11,1		Cell. proc
HEAR2702	*cyoA*	ubiquinol oxidase subunit II precursor	-3,8		Energy met
HEAR2244	*aspC*	Aspartate amùinotransferase (Transaminase)	-2,3		Aa biosynthesis
HEAR0126		Putative tRNA (guanine-N(7))methyltransferase	-2,0		Protein synthesis
HEAR0354	*frcA1*	Formyl coenzyme A transferase (Formyl-CoA transferase)	-2,5		Cent. Int. met
HEAR3133		Conserved hypothetical protein ; putative CYTOCHROME C BIOGENESIS PROTEIN	-2,1		Energy met
HEAR1131		putative glycosyl transferases group 1	-3,0		Cell env
HEAR1445	*psd*	phosphatidylserine decarboxylase	-2		Fatty acid
HEAR2919	*ctaC*	CYTOCHROME C OXIDASE subunit 2	-2,9		Energy met

HEAR2597		putative glycosyltransferase	-2,1		Cell env
HEAR0551	*cafA*	RNase G (ribonuclease G)	-2,4		Cell. proc
HEAR0405	*miaA*	tRNA delta(2)-isopentenylpyrophosphate tRNA-adenosine transferase	-2,9		Protein synthesis
HEAR2737	*pyrB*	aspartate carbamoyltransferase (ATCase)	-2,3		Pur. Pyr. Ns. Nts
HEAR1272	*hflC*	Protein HflC	-2,1		Protein fate
HEAR1826	*nuoA*	NADH-quinone oxidoreductase chain A (NADH dehydrogenase I, chain A) (NDH-1, chain A)	-2,1		Energy met
HEAR3126	*mrcA2*	penicillin-binding protein 1A	-2,1		
HEAR1343	*ecfK*	Putative bacterial surface antigen(D15)	-2,4		Cell env
HEAR1938		putative General secretion pathway protein, putative Mannose-sensitive agglutinin (MSHA) biogenesis protein	-3,2		Cell env
HEAR2791	*pilD*	type 4 prepilin-like proteins leader peptide processing enzyme (Protein secretion protein XCPA)[Includes: Leader peptidase (Prepilin peptidase); N-methyltransferase]	-2,8		Cell env
HEAR2071	*rpoE*	RNA polymerase sigmaE factor	-3,6		Reg. function
HEAR1760		Major facilitator superfamily (MFS-1) transporter	-2,6		Transport
HEAR2984	*slmA*	Putative regulatory protein, TetR family	-2,3		Reg. function
HEAR2739		putative holliday junction resolvase	-2,2		DNA met
HEAR1684		putative regulatory protein	-9,1		Reg. function
HEAR1683		putative permease	-2,1		Transport
HEAR2507		Putative carbonic anhydrase	-2,2		Cent. Int. met
HEAR2427		putative 3-oxoacyl-[acyl-carrier-protein] reductase	-2,4		Fatty acid
HEAR1644		CYTOCHROME C OXIDASE cbb3'-type SUBUNIT 1	-8,3		Energy met
HEAR1442		Putative 2-nitropropanedioxygenase	-10		Energy met
HEAR1650	*hemNB*	Oxygen-independent coproporphyrinogen III oxidase	-16,7		Biosynthesis cof
HEAR1651	*hemNA*	Oxygen-independent coproporphyrinogen III oxidase	-14,3		Biosynthesis cof
HEAR2426		Putative Major facilitator superfamily protein	-2,2		Transport
HEAR1950		Putative mannose-sensitive hemagglutinin a MshA-like	-2,5		Transport
HEAR0180	*lemA*	LemA	-2,1		Cell. proc
HEAR0408	*amiC*	N-acetylmuramoyl-L-alanine amidase	-2,7		Cell env
HEAR2060	*pdxJ*	Pyridoxine 5'-phosphate synthase (PNP synthase)	-3,2		Biosynthesis cof
HEAR2222		TatD related deoxyribonuclease	-2,9		DNA met
HEAR1652		Putative Nnrs protein	-3,8		Cent. Int. met
HEAR1389		putative shikimate transporter	-2,9		Transport

CLUSTER 4

HEAR0270		putative carboxymethylenebutenolidase		2,4	Cent. Int. met
HEAR0482	*aoxS*	Signal transduction protein involved in AoxAB regulation (AoxS)		3,1	Cell. proc
HEAR0483	*aoxR*	AoxR regulatory protein		3,3	Cell. proc
HEAR0656		putative ABC-type phosphonate transport system, periplasmic component		3,7	Transport
HEAR1655		hypothetical protein, metal binding domain	-7,1		Cell. proc
HEAR2541		putative NADPH dehydrogenase	-7,7		Energy met
HEAR2701	*cyoB*	ubiquinol oxidase subunit I	-3,7		Energy met
HEAR2884	*hprK*	HPr kinase/phosphorylase (HPrK/P) (HPr(Ser) kinase/phosphorylase)	-2,2		Reg. function
HEAR1794		putative transcriptional regulatory protein MerR family	-2,4		Reg. function
HEAR1648		cation transporting P-type ATPase ; probable copper exporting CopA-like	-3,7		Cell env
HEAR1137		putative UDP-glucose 4-epimerase	-2,6		Cell env
HEAR0937		Conserved hypothetical protein; putative ABC transporter	-2,1		Transport
HEAR2174		putative lipoprotein	-2,5		Cell env
HEAR2883	*ptsN*	Putative PTS system IIA like nitrogen regulatry protein PtsN	-2		Transport
HEAR1160		conserved hypothetical protein ; putative lipoprotein	-3,7		Cell env
HEAR3108		putative ABC-type tansport system, ATPase component	-2,6		Transport
HEAR2367	*adh*	Putative alcohol dehydrogenase (ADH)	-4,8		Cent. Int. met
HEAR2366		putative alcohol dehydrogenase (ADH)	-4,5		Cent. Int. met
HEAR0630		putative glycosyltransferase	-2,8		Cell env
HEAR2405		putative Biopolymer transport protein	-2,3		Transport
HEAR2698		Conserved hypothetical protein, putative cytochrome C biogenesis	-2,7		Biosynthesis cof
HEAR2877		Polysaccharide deacetylase	-2,2		Cent. Int. met
HEAR2258		putative Outer membrane protein OmpW-like	-20		Cell env
HEAR2404	*tolQ*	Protein TolQ	-2,3		Cell env
HEAR1135		Putative dTDP-4-dehydrorhamnose-3,5-epimerase	-2,1		Cell env
HEAR1515	*ackA*	Acetate kinase (Acetokinase)	-5		Cent. Int. met
HEAR2345	*phbB2*	AcetacetylCoA reductase	-2,2		Cell. proc
HEAR0116		putative DNA protecting protein DprA	-2,2		DNA met
HEAR2792	*coaE*	dephospho-CoA kinase	-4,2		Biosynthesis cof
HEAR3119	*dgt*	Deoxyguanosinetriphosphate triphosphohydrolase-	-2,6		Pur. Pyr. Ns. Nts

		like protein			
HEAR2745		Putative chemotaxis signal transduction protein CheW-like, putative PilI	-2,7		Cell. Proc
HEAR2365	*cspD1*	Cold shock protein CspD	-10,5		Cell. Proc
HEAR1316		Putative flagellar motor protein MotD	-2,5		Cell. Proc
HEAR1136	*rfbF*	Glucose-1-phosphate cytidylyltransferase (CDP-glucose pyrophosphorylase)	-2		Cell env
HEAR2940		putative CDP-diacylglycerol--serine O-phosphatidyltransferase partial	-2,3		Fatty acid
HEAR0737	*nirM*	cytochrome c-551 precursor	-2,0		Energy met
HEAR1641	*ccoP*	CYTOCHROME C OXIDASE cbb3-type, subunit III	-3,6		Energy met
HEAR1399		Phosphinothricin N-acetyltransferase	-2,1		Cell. Proc
HEAR1643	*fixO*	Cytochrome c oxidase cbb3-type, monoheme subunit	-4,2		Energy met
HEAR1654	*narL*	nitrate/nitrite response regulator protein	-3,2		Energy met
HEAR1642		putative cytochrome oxidase subunit III cbb3-type	-3,6		Energy met
HEAR0449	*aer*	aerotaxis receptor	-7,7		Signal transduction
HEAR1653		putative NITRATE/NITRITE SENSOR protein NarX	-7,7		Cell env
HEAR1514		Putative universal stress protein UspA	-9,1		Cell. Proc
HEAR1577		Putative universal stress protein UspA	-8,3		Cell. Proc
HEAR0585	*sbcB*	ExodeoxyribonucleaseI	-2		DNA met
HEAR0236		putative porin	-3,6		Transport
HEAR2202		putative phospholipase	-2,1		Cell. Proc
HEAR0813	*phaP*	Granule-associated protein Phasin	-7,1		Fatty acid

CLUSTER 5

HEAR0159		Putative Permease of the major facilitator superfamily		3,5	Transport
HEAR0476	*aoxD*	Cytochrome c-552 precursor (Cytochrome c552)		9,3	Cell. Proc
HEAR0486		ABC-type phosphate transport system, periplasmic component		126,7	Transport
HEAR0488		putative ABC-type phosphate transport system, permease component		84,9	Transport
HEAR0498	*sphX*	Phosphate-binding periplasmic protein precursor (PBP)		49,2	Transport
HEAR0580		putative DNA-polymerase/ribonuclease/exonuclease		2,6	DNA met
HEAR0784		Putative threonine efflux protein		4,4	Transport
HEAR0785		putative permease of the drug/metabolite transporter (DMT)		4,3	Transport
HEAR1108	*pstC2*	Phosphate transport system permease protein PstC		10,8	Transport
HEAR1109	*pstA2*	Phosphate transport system permease protein PstA		12,0	Transport
HEAR2560		Putative Mg2+ and Co2+ transporter		4,1	Transport
HEAR3213	*ptxC*	putative phosphonates transport system permease protein phnE-like		14,7	Transport
HEAR2334		Putative HSP20-like chaperone	-2,6		Cell. Proc
HEAR1780	*mdh*	malate dehydrogenase	-2,9		Cent. Int. Met
HEAR3134		Cytochrome c4	-2,5		Energy met
HEAR3048	*sspB*	stringent starvation protein B	-2,2		Cell. Proc
HEAR2763	*accC*	Biotin carboxylase (ACC)	-2,7		Fatty acid
HEAR3051	*petB*	Cytochrome b	-2,1		Energy met
HEAR2492		Putative biopolymer transport protein ExbB-like	-2,4		Transport
HEAR1348	*lpxB*	LipidA-disaccharide synthase	-5		Cell env
HEAR3052	*petA*	Ubiquinol-cytochrome c reductase iron-sulfur subunit (Rieske iron-sulfur protein) (RISP) (partial)	-2,6		Energy met
HEAR2379	*nrdR*	Transcriptional repressor NrdR	-2,8		Reg. Function
HEAR2398		putative cobalamin (vitamin B12) biosynthesis CblX protein	-2,3		Biosynthesis cof.
HEAR0377	*cutE*	apolipoprotein N-acyltransferase	-2,2		Cell env
HEAR2637	*pcnB*	poly(A) polymerase I	-2,6		DNA met
HEAR1129		putative polysaccharide biosynthesis protein	-2,6		Cell env
HEAR1350		putative tRNA/rRNA methyltransferase SpoU-like	-2,9		Protein synthesis
HEAR0368	*pdxA*	4-hydroxythreonine-4-phosphate dehydrogenase	-2,1		Biosynthesis cof
HEAR1138		putative glycosyltransferase	-2,6		Cell env
HEAR1273	*hflC*	Protein HflC	-2,5		Protein fate
HEAR1749	*hupB*	DNA-binding protein HU-beta, NS1 (HU-1)	-5,6		DNA met
HEAR2157		conserved hypothetical protein, putative phosphate transport regulator	-2,1		Reg. Function
HEAR0247		putative peptidase	-4,3		Protein fate
HEAR2738	*pyrR*	Bifunctional protein PyrR	-2,8		Reg. Function
HEAR0954	*cobT*	Nicotinate-nucleotide--dimethylbenzimidazole phosphoribosyltransferase	-2,6		Biosynthesis cof
HEAR1149	*galE2*	UDP-glucose 4-epimerase (Galactowaldenase) (UDP-galactose 4-epimerase)	-2,1		Cell env
HEAR2057	*uvrC*	UvrABC system protein C	-2,0		Cell. Proc
HEAR2085		putative Iron-sulfur cluster-binding protein, Rieske family	-2,0		Energy met
HEAR2322	*mltD*	Putative lytic transglycosylase	-2,1		Cell env
HEAR1071	*minE*	cell division topological specificity factor	-2,8		Cell. proc

HEAR1072	*minD*	Septum site determining protein MinD	-2,7		Cell. Proc
HEAR2761	*aroQ*	3-dehydroquinate dehydratase, type II	-2,6		Aa biosynthesis
HEAR1397	*holB*	DNA polymerase III, delta prime subunit	-2,3		DNA met
HEAR1001		putative TRAP decarboxylate transporter DctM subunit	-2,1		Transport
HEAR1073		Putative septum formationinhibitor MinC	-2,1		Cell. Proc
HEAR0975		conserved hypothetical protein ; putative segregation and condensation protein A	-2,3		Cell. Proc
HEAR2631		putative two-component sensor kinase	-2,3		Reg. Function
HEAR0556	*hemF*	coproporphyrinogen III oxidase, aerobic	-2,0		Biosynthesis cof
HEAR1530		Putative phage protein	-2,6		Mobile elements
HEAR2425		putative major facilitator superfamily protein	-2,6		Transport
HEAR0794		putative site-specific recombinase Gcr	-2,3		DNA met

CLUSTER 7

HEAR1156		Putative ABC-type Fe3+ transport system		2,4	Transport
HEAR3210		Transposase IS30 family		2,1	Mobile element
HEAR1744	*dadA*	D-amino acid dehydrogenase, small subunit	-3,5		Cent. Int. Met
HEAR0369	*ksgA*	S-adenosylmethionine-6-N',N'-adenosyl (rRNA) dimethyltransferase	-2,0		Cell. Proc
HEAR1800	*infC*	Translation initiation factor IF-3	-4		Protein synthesis
HEAR0170		Putative Rod shape-determining protein mreD	-3,0		Cell env
HEAR0974	*panC*	Pantoate--beta-alanine ligase	-2,2		Biosynthesis cof.
HEAR2061	*recO*	DNA repair protein recO (Recombination protein O)	-3,4		Cell. Proc
HEAR2187	*ftsB*	Cell division protein ftsB homolog	-2,3		Cell. Proc
HEAR0272		putative Zinc transporter ZIP	-3,3		Transport
HEAR2798	*secA*	preprotein translocase subunit SecA	-5		Transport
HEAR2963		putative outer membrane protein	-3,1		Transport
HEAR2708		Protein-L-isoaspartate (D-aspartate) O-methyltransferase	-2,9		Protein fate
HEAR2438		Pseudouridine synthase	-2,9		Protein synthesis
HEAR0968		putative threonine-phosphate decarboxylase (L-threonine-O-3- phosphate decarboxylase)	-2,9		Biosynthesis cof
HEAR3129	*lysA*	diaminopimelate decarboxylase (DAP decarboxylase)	-2,5		Aa biosynthesis
HEAR2867		conserved hypothetical protein; putative exporter protein	-2,6		Transport
HEAR2327	*fabI*	enoyl-[acyl-carrier-protein] reductase (NADH)	-2,4		Fatty acid
HEAR2638	*folK*	2-amino-4-hydroxy-6-hydroxymethyldihyropteridine pyrophosphokinase	-2,9		Biosynthesis cof
HEAR2087	*rluC*	Ribosomal large subunit pseudouridine synthase C	-2,0		Protein synthesis
HEAR2224	*lolC*	Lipoprotein releasing system transmembrane protein LolC	-2,3		Transport
HEAR0973		conserved hypothetical protein, putative porin	-2,6		Transport
HEAR1275	*purA*	adenylosuccinate synthetase	-2,1		Cent. Int. Met
HEAR0450	*rhlE1*	putative ATP-dependent RNA helicase	-2,9		Transcription
HEAR2074	*acpP*	acyl carrier protein (ACP)	-2		Fatty acid
HEAR1948		putative Mannose-sensitive agglutinin (MSHA) biogenesis protein MshD (pilus type IV)	-2,8		Cell env
HEAR0036		putative TonB-dependent siderophore receptor	-2,1		Transport
HEAR1947		putative MSHA biogenesis protein MshO	-2,6		Cell env
HEAR0459		putative glycosyl transferase	-2,8		Cell env
HEAR0407	*dedA*	Protein DedA	-2,1		Cell env

CLUSTER 9

HEAR0191		putative Phosphate starvation-inducible protein *psiF* precursor		4,7	Cell.proc
HEAR1180	*iorB*	Isoquinoline 1-oxidoreductase, beta subunit		2,3	Energy met
HEAR1413	*fdhB*	formate dehydrogenase iron-sulfur subunit		2,6	Energy met
HEAR1869	*fliS*	Flagellar protein		2,5	Cell. proc
HEAR1870		putative flagellar protein FliT		2,4	Cell. proc
HEAR1890		putative flagellar hook-associated protein 3 FlgL-like		2,2	Cell. proc
HEAR1903		putative negative regulator of flagellin synthesis (Anti-sigma-28 factor) FlgM-like		2,1	Cell. proc
HEAR2362		Conserved hypothetial protein, putative zinc finger domain	-5,6		Reg. fucntion
HEAR2700	*cyoC*	cytochrome o ubiquinol oxidase, subunit III	-2,9		Energy met
HEAR2542		putative nitroreductase	-5,6		Energy met
HEAR0315		Transposase of ISHar1, IS3	-2		Mobile elements
HEAR2358		Putative ABC transport syste, ATP binding protein	-3,3		Transport
HEAR2457		putative outer membrane lipoprotein; precursor SlyB-like	-6,7		Cell env
HEAR2543		putative Spermidine synthase	-2,4		Cent. Int. met
HEAR2354		conserved hypothetical protein ; putative membrane protein	-2,2		Transport
HEAR3124		Putative type IV fimbrial biogenesis protein PilN	-3,2		Cell env

HEAR1314	*fliA*	sigma fzctor for flagellar operon	-2,4		Cell. proc
HEAR1902	*flgA*	flagellar basal-body P ring formation protein FlgA precursor	-4,2		Cell. proc
HEAR2351		putative univeral stress protein UspA	-5,3		Cell. proc
HEAR2337		Putative transport associated protein	-5		Transport
HEAR1315	*motA2*	Motility protein A	-2,0		Cell. proc
HEAR2338		putative Universal stress protein UspA-like	-6,7		Cell. proc
HEAR0003	*dnaN*	DNA polymerase III, beta-subunit	-2		DNA met
HEAR1874	*fliE*	flagellar hook basal-body complex protein FliE	-2,4		Cell. proc
HEAR3123	*pilO*	Pilus assembly protein PilO precursor	-2,7		Cell env
HEAR1728		Putative ABC type branched amino acid transportsystems, periplasmic component	-2,0		Transport
HEAR3122		putative Tfp pilus assembly protein PilP	-2,3		Cell env
HEAR2340		Putative poly-beta-hydroxybutyrate polymerase	-3,3		Energy met
HEAR1513		Putative Universal stress protein UspA	-5,6		Cell. proc
HEAR0076	*cheW1*	Chemotaxis protein cheW	-2,4		Cell env
HEAR0654	*tar2*	methyl-accepting chemotaxis protein II, probable aspartate sensor	-4,8		Cell env
HEAR1901	*flgB*	flagellar basal-body rod protein FlgB	-4		Cell. proc
HEAR2746	*pilH*	twitching motility two-component response regulator transcription regulator protein PilH	-2,1		Cell. proc

CLUSTER 11

HEAR3216		Transposase IS30 family		2,1	Mobile element
HEAR2189	*pyrG*	CTP synthetase (UTP—ammonia ligase)	-2,1		Pur. Pyr. Ns. Nts
HEAR0796		putative cytochrome c	-2,4		Energy met
HEAR2491	*exbD*	putative biopolymer transport protein	-2,5		Transport
HEAR0795		Putative cytochrome c	-3,1		Energy met
HEAR0970	*cobQ*	cobyric acid synthase	-2,3		Biosynthesis cof.
HEAR2089	*rne*	Ribonuclease E (RNase E)	-2,5		DNA met
HEAR0384	*gdhA*	glutamate dehydrogenase, NADP-specific	-4,5		Aa biosynthesis
HEAR1011	*ppa*	inorganic pyrophosphatase (Pyrophosphate phospho- hydrolase) (PPase)	-2,2		Cent. Int. met
HEAR2434	*nusA*	transcription elongation protein NusA	-2,3		Reg. Function
HEAR2073	*fabF1*	3-oxoacyl-[acyl-carrier-protein] synthase II	-3,4		Fatty aicd
HEAR1271	*hflX*	GTP binding protein HflX	-2		Cell. proc
HEAR1236		putative RNA polymerase sigma factor	-2,3		Reg. function
HEAR1276		putative nucleotide phosphoribosyltransferase	-2,8		Cent. Int. met
HEAR2762	*accB*	Biotin carboxyl carrier protein	-2,4		Fatty acid
HEAR2173		Putative Metallo hydrolase/oxidoredustase superfamily	-2		Cell. proc
HEAR2709		putative rhodanese-related sulfurtransferase	-2,6		Cent. Int. met
HEAR0256	*ruvC*	Crossover junction endodeoxyribonuclease ruvC (Holliday junction nuclease ruvC) (Holliday juction resolvase ruvC)	-2,2		DNA met
HEAR2242		putative Uracil DNA glycosylase	-2,0		DNA met
HEAR2582		putative outer membrane protein OmpA	-2,4		Cell env
HEAR0966	*cobU*	bifunctional adenosylcobalamin biosynthesis protein CobU	-2,0		Biosynthesis cof
HEAR2850	*potB*	spermidine/putrescine transport system permease protein PotB	-2,6		Cell env
HEAR1949		putative MSHA pilin protein MshC	-2,5		Cell env
HEAR1040		Putative lytic transglycosylase	-3,0		Cent. Int. met
HEAR0955		putative cobalamin synthase CobS	-2,0		Biosynthesis cof
HEAR2740		conserved hypothetical protein, putative transcriptional regulator	-2,0		Reg. function

CLUSTER 12

HEAR0104		putative TRAP-type C4-dicarboxylate transport system, large permease component		2,2	Transport
HEAR0504		putative amino-acid metabolite efflux pump		8,9	Transport
HEAR0505		Putative Na+-dependent transporter		2,3	Transport
HEAR1103		putative L-asparaginase		3,0	Cent. Int. met
HEAR2113		transposase IS21 family (partial)		2,7	Mobile element
HEAR0345		putative fumarylacetoacetate (FAA) hydrolase	-2,4		Cent. Int. met
HEAR2598	*pdxH*	Pyridoxamine 5’ Phosphate oxidase	-8,3		Biosynthesis cof.
HEAR2353		ABC transport, ATP binding protein	-2,9		Transport
HEAR1210		putative signal transduction histidine kinase	-2,1		Reg. function
HEAR1253		putative ATP-dependent helicase	-2,0		DNA met
HEAR1516		putative phosphate acetyltransferase	-4,8		Cent. Int. met
HEAR2352		conserved hypothetical protein ; putative nitroreductase	-8,3		Energy met
HEAR1640		Putative ferredoxin	-2,3		Energy met
HEAR2364		putative DNA polymerase X	-2,2		DNA met
HEAR2448	*cspD2*	Cold scock like protein CspD	-2,3		Cell. proc
HEAR2744		Putative TWITCHING MOTILITY TRANSMEMBRANE PROTEIN PilJ-like	-2,3		Cell env
HEAR3346		putative Universal stress protein UspA	-11,1		Cell. proc
HEAR1647		Putative cytochrome oxidase maturation protein cbb3-type, putative Pill	-2,7		Energy met
